# Comparison of antibiotic resistant *Escherichia coli* obtained from drinking water sources in northern Tanzania: a cross-sectional study

**DOI:** 10.1186/s12866-016-0870-9

**Published:** 2016-11-03

**Authors:** Beatus Lyimo, Joram Buza, Murugan Subbiah, Woutrina Smith, Douglas R. Call

**Affiliations:** 1 Nelson Mandela African Institution of Science and Technology, 447, Arusha, Tanzania; 2 Paul G. Allen School for Global Animal Health, Washington State University, Pullman, WA 99164 USA; 3 One Health Institute, School of Veterinary Medicine, University of California, Davis, CA 95616 USA

**Keywords:** Antibiotic resistance, Water quality, Low-income country, Tanzania

## Abstract

**Background:**

Antimicrobial resistance (AMR) is a growing and significant threat to public health on a global scale. *Escherichia coli* comprises Gram-negative, fecal-borne pathogenic and commensal bacteria that are frequently associated with antibiotic resistance. AMR *E. coli* can be ingested via food, water and direct contact with fecal contamination.

**Methods:**

We estimated the prevalence of AMR *Escherichia coli* from select drinking water sources in northern Tanzania. Water samples (*n* = 155) were collected and plated onto Hi-Crome *E. coli* and MacConkey agar. Presumptive *E. coli* were confirmed by using a *uidA* PCR assay. Antibiotic susceptibility breakpoint assays were used to determine the resistance patterns of each isolate for 10 antibiotics. Isolates were also characterized by select PCR genotyping and macro-restriction digest assays.

**Results:**

*E. coli* was isolated from 71 % of the water samples, and of the 1819 *E. coli* tested, 46.9 % were resistant to one or more antibiotics. Resistance to ampicillin, streptomycin, sulfamethoxazole, tetracycline, and trimethoprim was significantly higher (15–30 %) compared to other tested antibiotics (0–6 %; *P* < 0.05). Of the β-lactam-resistant isolates, *bla*_TEM-1_ was predominant (67 %) followed by *bla*_CTX-M_ (17.7 %) and *bla*_SHV-1_ (6.0 %). Among the tetracycline-resistant isolates, *tet*(A) was predominant (57.4 %) followed by *tet*(B) (24.0 %). *E. coli* isolates obtained from these water sources were genetically diverse with few matching macro-restriction digest patterns.

**Conclusion:**

Water supplies in northern Tanzania may be a source of AMR *E. coli* for people and animals. Further studies are needed to identify the source of these contaminants and devise effective intervention strategies.

**Electronic supplementary material:**

The online version of this article (doi:10.1186/s12866-016-0870-9) contains supplementary material, which is available to authorized users.

## Background

Despite meeting the Millennium Development Goals for drinking water [1], 748 million mostly poor and marginalized people still lack access to quality drinking water. Of these almost a quarter (173 million) rely on untreated surface water *on a daily basis* and over 90 % live in rural areas [1]. Fecal waste from people and animals is a major source for water pollution, particularly in low-income countries. For instance, in 2012 approximately 1 billion people in the world did not have access to toilet facilities and instead used open and unsanitary places for defecation [1]. These communities also lack proper water supplies and depend heavily on untreated surface or shallow, unprotected water (river, ponds, and lakes) sources for consumption [2, 3]. These water sources run through agricultural lands and open places and thus are frequently exposed to human and animal wastes. Consuming these contaminated water increases the risk of exposure to enteric bacterial, viral and protozoan pathogens that could cause severe diseases in people [4]. Notably, there are several strains of *E. coli* that are pathogenic to both people and animals and that can cause disease ranging from self-limiting diarrhea to life-threatening hemolytic-uremic syndrome (HUS) and hemorrhagic colitis [5, 6].

Emergence and dissemination of antimicrobial-resistant (AMR) bacteria is considered the third-largest threat to global public health in the 21^st^ century [7]. AMR reduces the effectiveness of antibiotic treatment and thus leads to increased morbidity, mortality, and healthcare expenditures [8]. Commensal *E. coli* found in people and animals is considered a potential reservoir for AMR genes [9] and these genetic traits can be transferred to *E. coli* or to other bacteria found in people, animals, and in the environment [10]. Presumably, water sources contaminated with AMR bacteria is one contributing factor for the higher prevalence of antibiotic resistant bacteria from people living in low-income countries [11]. For instance, studies conducted in South Africa reported a high prevalence of antibiotic resistant *E. coli* from both “safe” (treated and closed) and unsafe (untreated and open) water sources [9, 12].

Tanzania is a low-income country in East Africa that is experiencing an exponential increase of human settlements around water sources [13]. A relatively small proportion of people living in the urban and peri-urban areas have access to what are considered safe (bore-well and taps) drinking water facilities. Consumption of water containing AMR bacteria is likely to increase the risk of disseminating antibiotic resistance and pathogenic bacteria. Nevertheless, the extent of this risk, if any, has not been well described.

In this study we assessed the prevalence of antibiotic resistant *E. coli* from surface waters in northern Tanzania. We also characterized a panel of isolates for the presence of select antibiotic resistance genes and assessed genetic diversity by using a macro-restriction assay with pulsed-field gel electrophoresis. In the long term, understanding the degree that surface waters are contaminated will help guide intervention strategies to reduce the risk of water-borne dissemination of pathogens and the dissemination of antibiotic resistance.

## Methods

### Study design

**Fig. 1:**
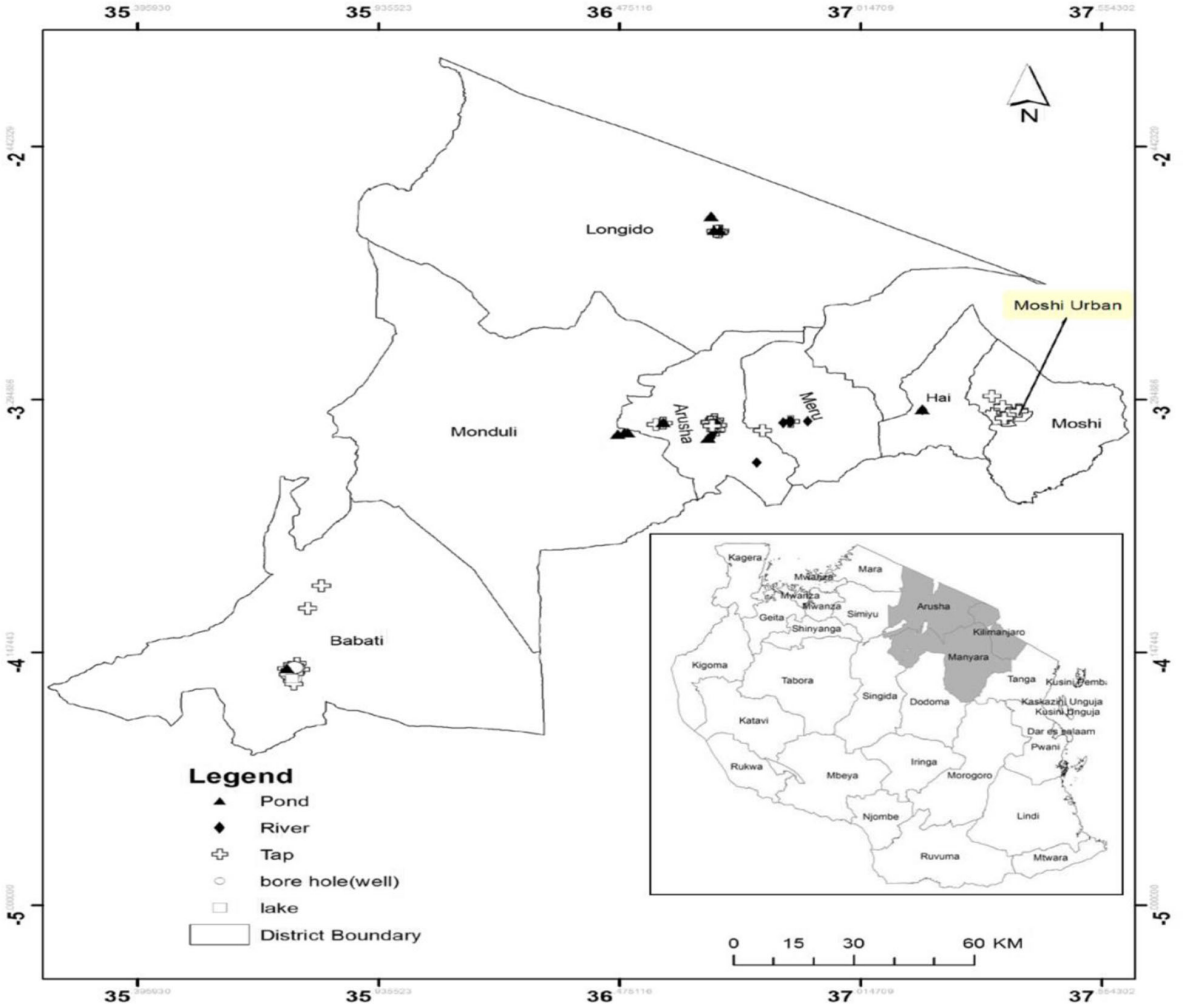
Distribution of water sampling locations in northern Tanzania

The study was cross-sectional and convenience sampling was used to collect 155 water samples in select water sources in northern Tanzania between March and September 2014 (Fig. 1). Permission was sought from the local authorities before collecting water from open (rivers, lakes, streams and ponds) and closed (taps and bore-wells) water resources located in two geographically and climatically distinct areas (dry-arid; Longido, Monduli and Simanjiro districts and lush-mountainous; Moshi, Arumeru and Arusha districts; Fig. 1). Ponds (surface rain water collected in manmade reservoirs) are shallow open bodies of water that are shared between animals (livestock and wildlife) and people living in the dry-arid regions. Rivers are the primary open surface waters that are used by people and animals living in the lush-mountainous regions. The number of households that use tap and bore-well water is relatively high in lush-mountainous compared to dry-arid regions. Water samples from the ponds were collected from localities near Maasai villages while the samples from rivers were collected from low risk-, moderate risk-, and high risk-stream segments of the same river in the vicinity of Arumeru (Nduruma, Tengeru) and Arusha (Temi) regions. The distance between the point where water was sampled and apparent human activities defined relative risk. If activities were observed >300 m from the sample collection point this was considered a “low risk” segment while moderate and high risk corresponded to within 50–300 m or within 50 m, respectively. Convenience sampling was used to collect tap water from Arusha, Arumeru, Longido, Moshi, and Monduli districts. Bore-well water samples were collected from Arusha, Arumeru, Monduli, and Simanjiro (Fig. 1).

### Sample collection and processing for the isolation of *E. coli*

Water samples were collected in 500-ml sterile bottles and transported in cooler boxes with ice packs to the laboratory for processing within 6 h (h) of collection. Water samples were analyzed using a standard membrane filtration technique with slight modification [14]. Briefly, 100 ml of each water sample from tap and well was filtered using a 47 mm membrane filter (Cellulose Nitrate filter, Sartorius Stedium Biotech GmbH, Göttingen, Germany) with a nominal pore size of 0.45 μm using a vacuum filtration system. To avoid clogging of membrane pores water samples (especially turbid) from pond, river and stream were serially diluted 1:100 before filtered using the standard membrane filtration technique as above. Following filtration each filter membrane was placed on a Chromogenic selective agar plate (Hi-Crome *E. coli* agar, Hi Media Laboratories Prt. Ltd, Mumbai, India) [15]. The agar plates were initially incubated at 37 °C for 4 h, followed by incubation for 16–22 h at 44 °C. In addition, the water samples (100 μl; undiluted) were directly plated onto MacConkey agar (Thermo Oxoid Remel, Lenexa, USA) using sterile glass beads and incubated overnight (~18 h) at 37 °C.

### Isolation and storage of *E. coli*

To determine the antibiotic susceptibility patterns of *E. coli* found in the water samples, up to 24 colonies/sample with morphology consistent with *E. coli* were picked from MacConkey agar plates using sterile toothpicks and inoculated into 96-well plates containing 150 μl of Luria-Bertani broth (Difco^TM^ LB Broth Lennox, Sparks, MD USA) per well. The plates were incubated overnight at 37 °C. After incubation sterile phosphate buffered glycerol was added into each well (15 % vol/vol, final volume of glycerol) and the plates were stored at -80 °C until shipping. For shipping, using a sterile 96-pin replicator (Boekel Scientific, Feasterville, USA), the pre-thawed 96-well plates containing cultures were sub-cultured into another 96-well plate containing 40 μl LB per well. These plates were incubated overnight at 37 °C. After incubation the plates were left open in a sterile incubator for drying between 32–35 °C for up to 24 h or until the cultures are completely desiccated. The completely desiccated plates were examined to confirm the absence of condensation and were then shipped to the Paul G. Allen School of Global Animal Health (Washington State University, Pullman, WA) for further analysis. Upon receipt 150 μl of LB broth was added to the desiccated wells and incubated overnight at 37 °C to recover the isolates. All the recovered isolates were tested for the presence of *uidA* gene specific for *E. coli* using a PCR [16].

### Antibiotic susceptibility testing

An antibiotic susceptibility breakpoint assay was used to estimate the prevalence of antibiotic-resistant *E. coli* from the study collection. For this assay, the following concentrations of antibiotics were used: amoxicillin/clavulanate potassium (AMC), 32/16 μg/ml (MP Biomedicals, Illkirch, France and Sigma-Aldrich, St Louis, MO, respectively); ampicillin (Amp), 32 μg/ml (Fisher Scientific, Fair Lawn, New Jersey); ceftazidime (Cfz), 8 μg/ml (Sigma-Aldrich); chloramphenicol (Chl), 32 μg/ml (Sigma-Aldrich); ciprofloxacin (Cip), 4 μg/ml (LKT Laboratories, Inc., St. Paul, MN); kanamycin (Kan), 64 μg/ml (Sigma-Aldrich); streptomycin (Str), 16 μg/ml (Fisher Scientific); sulfamethoxazole (Sul), 512 μg/ml (MP Biomedicals); tetracycline (Tet), 16 μg/ml (GTS, San Diego, CA); and trimethoprim (Tri), 8 μg/ml (MP Biomedicals). Selection of antibiotic concentrations was guided by the CLSI minimum inhibitory concentrations (MIC) for Enterobacteriaceae, except for streptomycin. The streptomycin concentration was determined by estimating the MIC for a susceptible K-12 strain of *E. coli* strain.

Breakpoint assays were conducted by preparing MacConkey agar with the indicated concentration of an antibiotic (one antibiotic per 150 mm plate). *E. coli* isolates were transferred onto the antibiotic containing media using a 96-pin replicator (Boekel Scientific, Fisher Scientific) from overnight culture in LB broth. After incubation at 37 °C for 24 h, growth was interpreted as resistant and no growth was interpreted as susceptible [17, 18]. *E. coli* strains K-12 (negative control, susceptible to all antibiotics tested) and H4H (positive control, *E. coli* strain harboring a ~148 kb plasmid that encodes resistance to 13 antibiotics including all of the antibiotics tested [19]) were used as reference strains for antibiotic susceptibility testing. The prevalence of antibiotic resistance was estimated by dividing the number of isolates resistant to a given antibiotic with the total number of isolates.

### Macro-restriction digest and pulsed-field gel electrophoresis (PFGE)

Thirty-seven *E. coli* isolates with >2 antibiotic resistance phenotypes from each water source were characterized by using Xbal macro-restriction digest assays with slight modifications from the PulseNet protocol [20]. Briefly, overnight culture was adjusted to an optical density of 1.4 using a spectrophotometer (610 nm) and 200 μl of the adjusted culture was augmented with proteinase K (10 μl at 20 mg/ml). Melted SeaKem Gold agarose (FMC, Rockland, ME, USA) was added and gently mixed before dispensing into plug molds (Bio-Rad, Hercules, CA) for 15 min. Plugs were incubated in ES buffer (0.5 M EDTA, pH 9.0, 1 % sodium lauroyl sarcosine) with proteinase K at 54 °C for 1 h and then washed 3X for 1 h in 1 M TE and 0.5 M EDTA buffer. Plugs were then treated with Xbal for 3 h in a water bath at 37 °C. Restriction fragments were then resolved by electrophoresis after inserting into 1 % SeaKem Gold agarose gels by using a CHEF DRIII apparatus (Bio-Rad, Hercules, CA). Gels were immersed in 0.5X Tris-borate–EDTA buffer and electrophoresis included initial switching time of 2.2 s and a final switching time of 54.2 s, 6 V and 120° angle. Fragments were resolved at 14 °C for 18 h. Control strains of *E. coli* O157:H7 and *Shigella* were included on every gel to improve accuracy of fragment size estimates. After electrophoresis the gels were stained with ethidium bromide for 20 min and destained 3X for 20 min each with deionized water. Gel images were collected using ChemiDoc XRS Gel Photo Documentation System (Bio-Rad) and analyzed using BioNumerics software version 4.0 (Applied Maths, Sint-Martens-Latem, Belgium). Cluster analysis was used to compare isolates by using the Unweighted Pair Group Method with Averages (UPGMA) with 1 % tolerance and a 0.5 % optimization setting based on Dice coefficients.

### Determining the prevalence of beta-lactam and tetracycline resistant *E. coli* genotypes

To estimate the prevalence of select antibiotic resistance genes, DNA templates of beta-lactam (Amp, AMC and Cfz) and tetracycline-resistant *E. coli* isolates were prepared by pelleting 2 ml of overnight culture at 12,000 × *g* for 10 min. The pellets were then re-suspended with 200 μl of nanopure water and boiled for 10 min in a heat block. The boiled suspensions were centrifuged briefly and 2 μl of the supernatant was used as DNA template for PCR assays. PCR assays were used to test for beta-lactamase encoding gene (*bla*_TEM-1_, *bla*_SHV-1_, and *bla*_CTX-M_) and two tetracycline resistance genes [*tet*(A) and *tet*(B)]. A total of 143 and 364 isolates (from both open and closed water sources) were tested for *bla*_TEM-1_, *bla*_SHV-1_, and *bla*_CTX-M_ and two tetracycline resistance genes *tet*(A) and *tet*(B), respectively. Positive (*E. coli* H4H) and negative (*E. coli* K12) control strains were included with each set of PCR tests. Reactions were performed using a DNA thermocycler (C1000 Touch Thermal Cycler, Bio-Rad) with specific primers (Table 1).

**Table 1:** Primers sequences used in this study

Primer	Nucleotide sequence	Reference
uidA	LGO_uidA F-5′-GACCCACACTTTGCCGTAAT-3′	This study
	LGO_uidA R-5′-AGTCTGGATCGCGAAAACTG-3′	
CTX-M	F- 5′-TTTGCGATGTGCAGTACCAGTAA-3′	[22]
	R-5′-CGATATCGTTGGTGGTGCCATA-3′	
CTX-M-1 group	F-5′-GGT TAA AAA ATC ACT GCG TC-3′	[22]
	R-5′-TTG GTG ACG ATT TTA GCC GC-3′	
CTX-M-2 group	F- 5′-ATG ATG ACT CAG AGC ATT CG-3′	[22]
	R-5′-TGG GTT ACG ATT TTC GCC GC-3′	
CTX-M-9, Toho-2 group	F-5′-ATG GTG ACA AAG AGA GTG CA-3′	[22]
	R-5′-CCC TTC GGC GAT GAT TCT C-3′	
*bla*_SHV_	F-5′-AGGATTGACTGCCTTTTTG-3′	[41]
	R-5′-ATTTGCTGATTTCGCTCG-3′	
*bla*_TEM_	F-5′-ATCAGCAATAAACCAGC-3′	[41]
	R-5′-CCCCGAAGAACGTTTTC-3′	
*tet*(A)	F-5′-GAAACCCAACATACCCCTGA-3′	This study
	R-5′-GAAGCTGAGCGGGTTGAGAG-3′	
*tet*(B)	F-5′-GTTCGACAAAGATCGCATTG-3′	This study
	R-5′-TCTGTATTATCACGTGTATTTTTGG-3′	

PCR reactions were performed in a total volume of 25 μl that included 2 μl DNA template, 12.5 μl master mix (ThemoFisher Scientific), 2 μl of each primer (10 μM) and 8.5 μl of PCR grade water. The following PCR running conditions were used for *bla*_TEM-1_: initial denaturation at 95 °C for 3 min, followed by 35 cycles of denaturation at 95 °C for 1 min, annealing at 63 °C for 1 min and extension at 72 °C for 1 min and final extension at 72 °C for 10 min. For *bla*_SHV-1_ PCR the conditions included initial denaturation at 94 °C for 30 s, followed by 32 cycles of denaturation at 94 °C for 30 s, annealing at 54 °C for 30 s and extension at 72 °C for 1 min and a final extension at 72 °C for 10 min. For *tet*(A) and *tet*(B) PCR the conditions included denaturation was at 95 °C for 5 min, followed by 30 cycles of denaturation at 95 °C for 30 s, 55 °C for 30 s and extension at 72 °C for 30 s and a final extension at 72 °C for 2 min [21, 22]. For CTX-M PCR the conditions included initial denaturation at 95 °C for 2 min followed by 30 cycles of denaturation at 95 °C for 15 s, annealing at 52 °C for 15 s, extension at 72 °C for 30 s and a final extension at 72 °C for 7 min [21]. Gel electrophoresis (1.5 % agarose) was used to analyze PCR products and a 1-kb DNA ladder (Gene ruler 1 Kb, Life Technologies) was used as a size standard. CTX-M grouping (group 1, group 2 and group 9) [22] was evaluated for all isolates that were positive for the CTX-M marker by using bi-directional DNA sequencing of CTX-M PCR amplicons (Functional Bioscience, Madison, WI). Sequencher (ver 5.0) software was used to process DNA traces, and the sequences were then compared with the reported sequences from GeneBank by using the BLAST utility (http://blast.ncbi.nlm.nih.gov/Blast.cgi).

### Statistical analysis

Descriptive statistics were used to estimate the prevalence of antibiotic resistance and a student’s *t*-test was used to analyze the mean of resistant isolates and resistance genes. MANOVA was used to compare the prevalence within and between groups where *P* ≤ 0.05 was considered significant (SPSS Inc., Chicago, IL, USA). Between-group statistical comparisons were adjusted for compounding error by using a Bonferroni adjustment to assess significance [*P* ≤ 0.05/(number of tests)]. CLC Main Workbench (CLC Bio Aarhus, Denmark) was used for sequence alignment.

## Results

A total of 155 water samples were collected from open (*n* = 93) and closed (*n* = 62) drinking water sources in dry-arid (*n* = 40) and lush-mountainous (*n* = 115) regions of northern Tanzania (Table 2). Presumptive *E. coli* was detected from 110 of 155 (71 %) water samples and 100 % of 1819 tested isolates were positive for the *uidA* PCR marker. Between dry-arid and lush-mountainous areas, we recovered an average of 11.2 and 11.9 *E. coli* isolates per sample, respectively (Table 2).

**Table 2:** Distribution of samples and antimicrobial resistant (AMR) *E. coli* in two different regions of northern Tanzania

Regions	District	Source	Nature	Samples	*Number of E. coli* isolates	AMR *E. coli*	% AMR *E. coli*
Dry-arid							
	Longido	Tap	Closed	7	9	5	55
	Monduli	Pond	Open	11	220	64	29
		River	Open	2	12	5	41
		Tap	Closed	8	44	30	68
	Simanjiro	Pond	Open	6	83	21	25
		River	Open	2	36	11	30.5
		Stream	Open	1	1	1	100
		Well	Closed	3	42	24	57
		Total		40	447	161	
Lush-mountainous							
	Arumeru	River	Open	39	672	378	56
		Stream	Open	1	26	25	96
		Tap	Closed	6	109	33	30
		Well	Closed	1	2	0	0
	Arusha	Pond	Open	2	6	5	83
		River	Open	12	190	70	37
		Stream	Open	7	71	41	58
		Tap	Closed	15	196	91	46
		Well	Closed	4	10	4	40
	Moshi	Pond	Open	3	4	1	25
		River	Open	4	10	2	20
		Stream	Open	3	24	16	66
		Tap	Closed	18	52	28	54
		Total		115	1,372	694	

Distribution of samples and antimicrobial resistant (AMR) *E. coli* isolated from open- and closed-water sources located in two different regions of northern Tanzania

### Prevalence of antibiotic-resistant *E. coli*

Antibiotic susceptibility testing demonstrated that at least 46.9 % (854 of 1819) of *E. coli* were resistant to one or more antibiotics. With ten antibiotics tested there was a possibility of detecting 2^10^ = 1024 different combinations of resistant phenotypes, of which we detected 104 with 99 including >1 resistance phenotype (Table 3, Additional file 1: Table S1). Isolates that were resistant to nine out of ten antibiotics were found in pond water from the dry-arid region (AmpAMCCfzCipChlStrSulTetTri) and one isolate that was resistant to seven antibiotics was found from a lush-mountainous tap water sample (Table 3).

**Table 3:** Percentage of multidrug resistant *E. coli* isolates obtained from drinking-water sources

Sources (number of isolates tested)	Number and (%) of resistance for multiple antibiotics		
	2–3	4–6	≥7 *P*
Dry-arid (447)	38 (8.5)	48 (10.7)	10 (2.2)
Open sources (352)	27 (7.6)	39 (11.2)	9 (2.5) 0.199
Closed sources (95)	15 (15.38)	7 (7.7)	0
Lush-mountainous (1,372)	182 (13.3)	189 (13.8)	23 (1.7)
Open sources (1,010)	147 (14.6)	145 (14.4)	9 (0.9) 0.17
Closed sources (362)	38 (10.6)	44 (12.04)	15 (4.2)

Antibiotics tested included ampicillin, amoxicillin/clavulanic acid, ceftazidime, ciprofloxacin, chloramphenicol, kanamycin, streptomycin, sulfamethoxazole, tetracycline, and trimethoprim

**Fig. 2:**
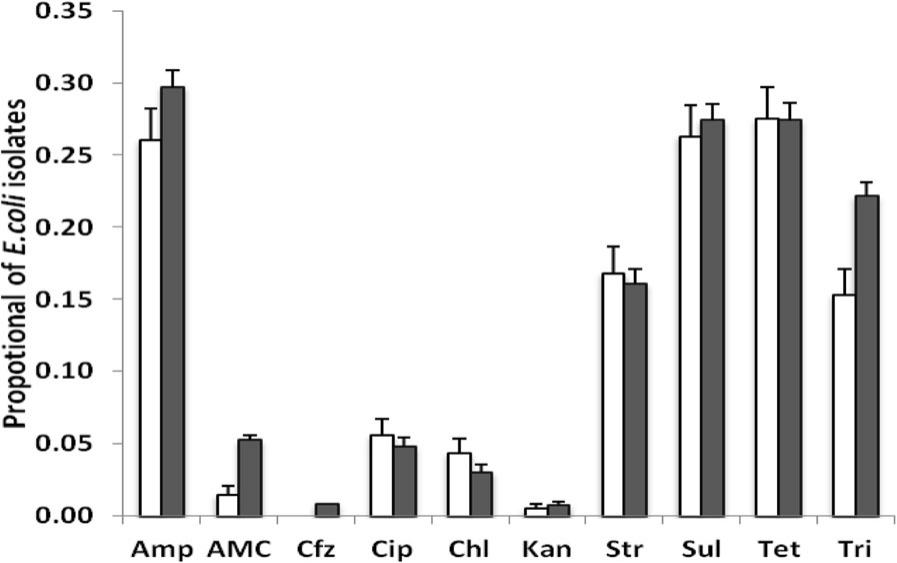
Proportion of antibiotic-resistant *E. coli* isolates recovered from closed (*white*) and open (*black*) water sources

The mean proportion of *E. coli* isolates resistant to ampicillin, streptomycin, sulfamethoxazole, tetracycline and trimethoprim was significantly higher (15–30 %) compared to other tested antibiotics (0–6 %; *P* < 0.05). Resistance to these antibiotics occurred alone or in association with other antibiotics for all of the tested waters. There was no statistical difference between the prevalence of antibiotic resistance in closed and open water sources, with the exception of the prevalence of trimethoprim resistance (Fig. 2).

### Prevalence of β-lactam and tetracycline resistance genes

**Fig. 3:**
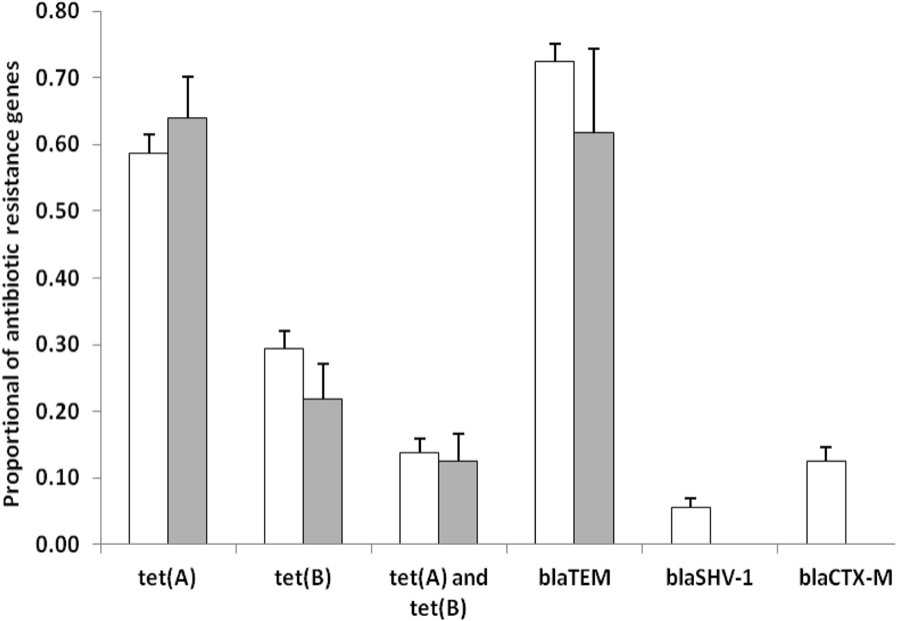
Proportion of antibiotic resistance genes in open (*white*) and closed (*black*) water sources

Out of 143 Amp-resistant *E. coli* isolates, there was no significant difference in the distribution between open and closed water isolates for *bla*_TEM -1_, *bla*_CTX-M_ and *bla*_SHV-1_ (Kruskal-Wallis test, *P* > 0.05) (Fig. 3). Among the 16 isolates positive for *bla*_CTX-M,_ fifteen were *bla*_CTX-M15_ and one was *bla*_CTX_-_M79_ (all from open water sources). Among the β-lactam-resistant isolates, *bla*_TEM-1_ was predominant (67 %) followed by *bla*_CTX-M_ (17.7 %) and *bla*_SHV-1_ (6.0 %). Among the 364 tetracycline-resistant *E. coli* isolates, the prevalence of *tet*(A) and *tet*(B) genotypes was equal for both open and closed water, respectively (Wilk’s Lambda = 0.95, *F* = 0.72, *P* = 0.54) (Fig. 3). Overall prevalence in tetracycline-resistant isolates, *tet*(A) was predominant (57.4 %) followed by *tet*(B) (24.0 %).

### Macro-restriction enzyme results

**Fig. 4:**
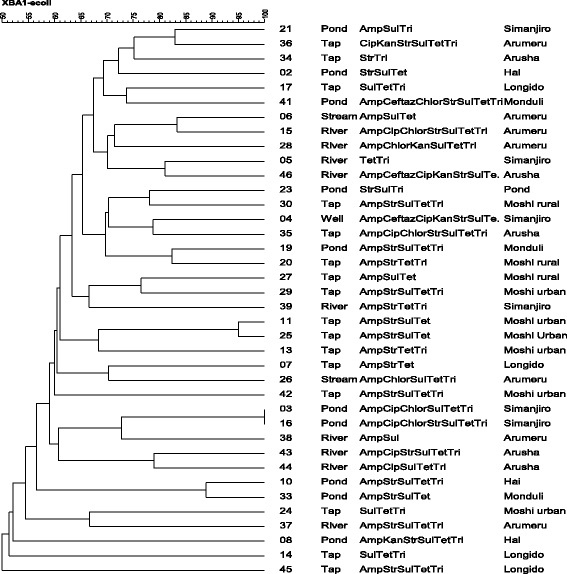
Dendrogram from macro-restriction digest assays for multidrug-resistant *E. coli* isolates from different surface water

Thirty-seven *E. coli* isolates with >2 antibiotic resistance phenotypes obtained from different water sources were genotyped by macro-restriction assays. A significant degree of genetic diversity was evident from these assays (Fig. 4). There were a few isolates (4 %) from the same water source that clustered into one group or in adjacent clusters. For instance, isolates #3 and #16 (pond water isolates from Simanjiro district) were clustered in the same group, while isolates #11, #25 and #13 (tap water isolates from Moshi urban area) were clustered together (Fig. 4) consistent with genetic similarity (Fig. 4).

## Discussion

We assessed the presence, genetic similarity, and distribution of AMR *E. coli* to determine if there might be a risk of transmission to people and animals. *E. coli* was isolated from 71 % of the water samples, and of 1819 *E. coli* tested, 46.9 % were resistant to one or more antibiotic. Some of the sampled waters are shared between people and animals and can easily be contaminated with fecal bacteria. Other studies have reported the presence of antibiotic-resistant *E. coli* from surface waters. For example, Nontongana et al., [23] reported that *E. coli* recovered from the Kat river in South Africa were resistant to ampicillin (98 %), tetracycline (13 %) and streptomycin (8 %). Dolejská et al., [24] reported that, on average, 17 % of surface water *E. coli* isolates were resistant to one or more antibiotics in the Czech Republic.

For the present study, the prevalence of resistance to ampicillin, streptomycin, sulfamethoxazole, tetracycline and trimethoprim was significantly higher than other tested antibiotics in both open and closed water sources. These five resistance phenotypes have been reported as relatively prominent for clinical isolates obtained from children (<5 years of age) in Tanzania [25] and this pattern of multidrug resistance has been reported elsewhere in both hospital and environmental settings [23, 26–28]. Presumably, the dissemination of these resistance traits is related to the spread of mobile genetic elements such as plasmids that are widely distributed among enteric bacteria [29]. In a separate study we characterized the plasmids from a panel of 31 isolates that were isolated from our water sample survey [15]. Plasmid typing (PCR) showed that these isolates harbored at least eleven plasmid replicon types (IncI1, FIC, P, FIIA, A/C, FIB, FIA, H12, K/B B/O, and N) and the filter-mating conjugation experiments indicated that these plasmids were transmitted to the recipient hosts at a rate ranged between 10^−1^ and 10^−7^ [15].

We detected considerable genetic variation among antibiotic-resistant isolates of *E. coli* from multiple water sources, which probably should not be unexpected given the degree of genetic resolution that is afforded by macro-restriction digest assays. Nevertheless, two isolates were indistinguishable (#3 and #16) suggesting that clonal dissemination may contribute to the dissemination of resistant bacteria and resistance genes.

Importantly, we found isolates that are resistant to ciprofloxacin from all types of water sources, although the prevalence of ciprofloxacin-resistant *E. coli* (5.3 %) was less than other studies that isolated *E. coli* from sewage and treatment ponds [30]. Ciprofloxacin, like other antibiotics in Tanzania, can be purchased easily from many drug shops without a prescription. This broad-spectrum antibiotic is frequently used to treat urinary-tract infections that are also commonly resistant to the first line of antibiotics such as amoxicillin, cotrimoxazole [31]. In Tanzania, resistance to members of first and second-line antibiotics has been documented in children and pregnant women with *E. coli* infection [31]. Therefore, caution should be taken to avoid indiscriminate and inappropriate use of antibiotics especially in developing countries like Tanzania where these drugs are often sold without prescription, contrary to drug regulation [32].

We also documented a high prevalence of the *bla*_TEM-1_ and *tet*(A) genes, which is similar to reports from India and Thailand [33, 34]. *Bla*_TEM-1_ is widely distributed by plasmids and encodes a clinically significant, broad spectrum β-lactamase that hydrolyzes many β-lactams in addition to many penicillins. Christopher et al., [25] reported that 100 % of *E. coli* clinical isolates from children in Tanzania were resistant to ampicillin and these may have harbored *bla*_TEM-1_. The high prevalence could also be due to higher utilization of beta-lactams antibiotic classes in many areas in Tanzania [35]. Moreover *tet*(A), which is plasmid mediated, encodes an active efflux pump that functions against all tetracycline drugs.

We found that *E. coli* collected from both closed and open water sources harbored *tet*(B) resistance genes, but we only detected *bla*_CTX-M79_ and *bla*_CTX-M15_ genes from open water sources. *Tet*(B) confers resistance to the second generation of tetracycline (e.g., minocycline) [36] while *bla*_CTX-M15_ exerts hydrolytic activity against ceftazidime, a third-generation cephalosporin that is frequently used in people [37]. The detection of *bla*_CTX-M15_ in water sources in northern Tanzania is consistent with previous reports of its widespread distribution [38]. *Bla*_CTX-M79_ has been isolated from people and farmed fish in China, and from cattle in the USA [39]. In Tanzania, we believe that this is the first report of *bla*_CTX-M79_ from *E. coli* water isolates*.* Emergence and dissemination of resistant bacteria for extended-spectrum cephalosporins in drinking water should be a point of concern to physicians and health officers who manage infections in both hospital and community settings [40].

## Conclusion

We report detection of a relatively high prevalence of ampicillin, tetracycline, trimethoprim, sulpamethoxazole and streptomycin resistant *E. coli* (15–30 %) in all sources of water commonly used by people in northern Tanzania. Because of shared-access, it is likely that livestock and people are exposed to AMR bacteria on a daily basis in communities where water treatment options are limited. Further work is needed to determine if these exposures lead to negative health outcomes.

## Additional file

Additional file 1: Table S1. Resistance pattern (104 different phenotypes). (DOC 134 kb)

## Abbreviations

AMR: Antimicrobial resistance
CLSI: Clinical and Laboratory Standards Institute
CTX-M: Cefotaxime- Munich
DNA: Deoxyribonucleic acid
ESBL: Extended spectrum β-lactamase
MANOVA: Multivariate Analysis of Variance
MDR: Multidrug resistance
PCR: Polymerase chain reaction
PFGE: Pulsed field gel electrophoresis
WHO: World Health Organization
