# Supplementary material for: Comparison of antibiotic resistant Escherichia coli obtained from drinking water sources in northern Tanzania: a cross-sectional study
Source: BMC Microbiol. 2016 Nov 3;16:254. doi: 10.1186/s12866-016-0870-9 (PMC5094041; doi:10.1186/s12866-016-0870-9)
Supplement: Additional file 1: Table S1. — Resistance pattern (104 different phenotypes). (DOC 134 kb) [file 12866_2016_870_MOESM1_ESM.doc]

Additional file 1: Table S1: **Resistance pattern (104 different phenotypes**)

|  | | |
| --- | --- | --- |
| **Antibiotic resistance phenotype** | Frequency | Percent |
| **Susceptible** | 971 | 53.1 |
| **Amp** | 130 | 7.1 |
| **AmpAmp/ClvStrSulTetTri** | 1 | .1 |
| **AmpAmx/Clv** | 8 | .4 |
| **AmpAmx/ClvCeftazChlorStrSulTetTri** | 1 | .1 |
| **AmpAmx/ClvCeftazCipChlor** | 1 | .1 |
| **AmpAmx/ClvCeftazCipChlorStrSulTetTri** | 2 | .1 |
| **AmpAmx/ClvCeftazCipChloStrSulTetTri** | 1 | .1 |
| **AmpAmx/ClvCeftazSul** | 1 | .1 |
| **AmpAmx/ClvChlorStrSulTetTri** | 1 | .1 |
| **AmpAmx/ClvCipChlorStrTetTri** | 1 | .1 |
| **AmpAmx/ClvCipChlorSulTri** | 1 | .1 |
| **AmpAmx/ClvCipSul** | 1 | .1 |
| **AmpAmx/ClvKanStrSulTetTri** | 1 | .1 |
| **AmpAmx/ClvStr** | 2 | .1 |
| **AmpAmx/ClvStrSul** | 1 | .1 |
| **AmpAmx/ClvStrSulTetTri** | 4 | .2 |
| **AmpAmx/ClvStrSulTri** | 4 | .2 |
| **AmpAmx/ClvStrTet** | 1 | .1 |
| **AmpAmx/ClvStrTetTri** | 1 | .1 |
| **AmpAmx/ClvSul** | 2 | .1 |
| **AmpAmx/ClvSulTet** | 3 | .2 |
| **AmpAmx/ClvSulTetTri** | 3 | .2 |
| **AmpAmx/ClvTri** | 1 | .1 |
| **AmpCeftazCipKanStrSulTetTri** | 3 | .2 |
| **AmpCeftazCipKanTetTri** | 2 | .1 |
| **AmpCeftazCipSulTetTri** | 1 | .1 |
| **AmpChlorKanStrSulTetTri** | 1 | .1 |
| **AmpChlorKanSulTetTri** | 1 | .1 |
| **AmpChlorStrSulTetTri** | 4 | .2 |
| **AmpChlorStrTet** | 1 | .1 |
| **AmpChlorStrTetTri** | 1 | .1 |
| **AmpChlorSulTetTri** | 4 | .2 |
| **AmpChlorSulTri** | 1 | .1 |
| **AmpChlorTetTri** | 1 | .1 |
| **AmpChlorTri** | 1 | .1 |
| **AmpCipChlorStrSul** | 1 | .1 |
| **AmpCipChlorStrSulTetTri** | 20 | 1.1 |
| **AmpCipChlorStrTet** | 1 | .1 |
| **AmpCipChlorStrTetTri** | 1 | .1 |
| **AmpCipChlorSulTet** | 1 | .1 |
| **AmpCipChlorSulTetTri** | 7 | .4 |
| **AmpCipChlorTri** | 1 | .1 |
| **AmpCipStrSul** | 1 | .1 |
| **AmpCipStrSulTet** | 2 | .1 |
| **AmpCipStrSulTetTri** | 6 | .3 |
| **AmpCipStrSulTri** | 2 | .1 |
| **AmpCipStrTetTri** | 1 | .1 |
| **AmpCipSulTetTri** | 8 | .4 |
| **AmpCipTet** | 3 | .2 |
| **AmpKanStrSulTet** | 1 | .1 |
| **AmpKanStrSulTetTri** | 9 | .5 |
| **AmpStr** | 1 | .1 |
| **AmpStrSul** | 7 | .4 |
| **AmpStrSulTet** | 32 | 1.7 |
| **AmpStrSulTetTri** | 65 | 3.7 |
| **AmpStrSulTri** | 18 | 1.0 |
| **AmpStrTet** | 3 | .2 |
| **AmpStrTetTri** | 17 | .9 |
| **AmpStrTri** | 3 | .2 |
| **AmpSul** | 25 | 1.4 |
| **AmpSulTet** | 11 | .6 |
| **AmpSulTetTri** | 48 | 2.7 |
| **AmpSulTri** | 14 | .8 |
| **AmpTet** | 15 | .8 |
| **AmpTetTri** | 6 | .3 |
| **AmpTri** | 5 | .3 |
| **Amx/Clv** | 24 | 1.4 |
| **Amx/Clv Str** | 2 | .1 |
| **Amx/ClvAmp** | 2 | .1 |
| **Amx/ClvCipSulTetTri** | 1 | .1 |
| **Amx/ClvStr** | 1 | .1 |
| **Amx/ClvSul** | 1 | .1 |
| **Amx/ClvSulTetTri** | 3 | .2 |
| **Amx/ClvTet** | 3 | .2 |
| **ChlorStrSulTetTri** | 2 | .1 |
| **ChlorStrTet** | 1 | .1 |
| **ChlorSulTetTri** | 1 | .1 |
| **Cip** | 3 | .2 |
| **CipChlorStrSulTetTri** | 1 | .1 |
| **CipKanStrSulTetTri** | 1 | .1 |
| **CipStrSulTet** | 3 | .2 |
| **CipStrSulTetTri** | 1 | .1 |
| **CipStrTetTri** | 2 | .1 |
| **CipSulTet** | 1 | .1 |
| **CipSulTetTri** | 8 | .4 |
| **CipTet** | 1 | .1 |
| **CipTetTri** | 1 | .1 |
| **Clv/ClvAmp** | 1 | .1 |
| **KanStrTet** | 2 | .1 |
| **Str** | 9 | .5 |
| **StrSul** | 2 | .1 |
| **StrSulTet** | 18 | 1.0 |
| **StrSulTetTri** | 16 | .9 |
| **StrSulTri** | 3 | .2 |
| **StrTet** | 11 | .6 |
| **StrTetTri** | 5 | .3 |
| **StrTri** | 1 | .1 |
| **Sul** | 50 | 2.8 |
| **SulTet** | 12 | .7 |
| **SulTetTri** | 33 | 1.9 |
| **SulTri** | 6 | .3 |
| **Tet** | 70 | 3.9 |
| **TetTri** | 10 | .6 |
| **Tri** | 8 | .4 |
| **Total** | 1819 | 100.0 |
